# New Insights into the Pharmacological Effects and Potential Health Benefits of Volatile Phenylpropenes

**DOI:** 10.3390/molecules31193400

**Published:** 2026-09-24

**Authors:** Simona-Codruța Hegheș, Oliviu Vostinaru, Anamaria Apan, Cristina Pop, Irina Iaru, Cristina Mogoșan, Mozaniel Santana de Oliveira, Oana Mîrza, Lorena Filip

**Affiliations:** 1 Department of Drug Analysis, Iuliu Hatieganu University of Medicine and Pharmacy, 400349 Cluj-Napoca, Romania; cmaier@umfcluj.ro; 2 Department of Pharmacology, Physiology and Physiopathology, Iuliu Hatieganu University of Medicine and Pharmacy, 400349 Cluj-Napoca, Romania; anamaria.apan@umfcluj.ro (A.A.); cristina.pop.farmacologie@gmail.com (C.P.); cazacuirina16@gmail.com (I.I.); cmogosan@umfcluj.ro (C.M.); 3 Laboratory of Pharmacology of Inflammation and Behavior, Federal University of Para, Belem 66075-900, PA, Brazil; mozaniel@ufpa.br; 4 Department of Bromatology, Hygiene, Nutrition, Iuliu Hatieganu University of Medicine and Pharmacy, 400349 Cluj-Napoca, Romania; oana.stanciu@umfcluj.ro (O.M.); lfilip@umfcluj.ro (L.F.)

**Keywords:** phenylpropenes, antimicrobial, anti-biofilm, antitumoral, anti-inflammatory, antidiabetic

## Abstract

Phenylpropenes are a significant category of plant volatile compounds that play a crucial role in enhancing the flavor of a variety of herbs and spices. They are a subclass of phenylpropanoid compounds, being important constituents in several plant species like basil, clove, cinnamon, anise, sassafras, nutmeg, and fennel. The most common molecules from the phenylpropene group are represented by anethole, chavicol, cinnamaldehyde, estragole, eugenol, myristicin (methoxy-safrole), and safrole. Beyond their culinary and cosmetic applications, phenylpropenes have been found to have multiple pharmacological effects, including being antimicrobial, antitumoral, antioxidant, anti-inflammatory, antidiabetic, and hypolipemic. This review is focused on the presentation of the latest scientific data resulting mainly from preclinical in vivo and in vitro studies which investigated the pharmacological effects of phenylpropenes, highlighting also the potential health benefits of phenylpropenes in several infectious, inflammatory, cardiovascular, and metabolic diseases. Mechanistic aspects regarding their biological effects and additional safety data are also reviewed.

## 1. Introduction

Volatile phenylpropenes are a subclass of phenylpropanoids, natural compounds with significant roles in plant development, serving as components of cell walls, protecting the plants from UV radiation, or playing defensive roles against various pathogens [1,2,3]. They are an important category of plant secondary metabolites produced via a cascade of enzymatic processes, initiating with the transformation of phenylalanine through the shikimate pathway [4].

Volatile phenylpropenes are widely present in essential oils, contributing to the flavor and aroma of multiple herbs and spices and also functioning as floral attractants for pollinators [5]. They are key chemical constituents in several economically important plant species like basil, clove, cinnamon, sassafras, nutmeg, and fennel. The most common volatile phenylpropenes are represented by anethole, chavicol, cinnamaldehyde, estragole, eugenol, myristicin (methoxy-safrole), and safrole [1,5]. Large amounts of volatile phenylpropenes can be obtained by direct plant extraction, chemical synthesis, and also by microbial engineering [6].

Phenylpropene-rich essential oils have been used in food recipes since antiquity, and in modern times, volatile phenylpropenes are regarded as high-value molecules for the agricultural, cosmetic, and biomaterials industries [7]. In recent decades, the pharmaceutical industry has also shown increased interest in their biological effects with regard to widespread resistance to current antimicrobial drugs and the growing incidence of inflammatory and metabolic diseases, which warrant the development of adjuvant therapeutic solutions. Thus, several lines of research have revealed that volatile phenylpropenes possess significant pharmacological activities, including antimicrobial, antitumoral, antioxidant, anti-inflammatory, and analgesic effects, but also hypoglycemic and lipid-lowering properties [1,7,8].

The versatile molecular structure of volatile phenylpropenes can interact with multiple biological targets in the human organism like enzymes, receptors, and ion channels, enabling them to exert protective effects in several inflammatory, cardiovascular, and metabolic diseases or cancer, with the potential to become successful adjuvants or even drug candidates with possible clinical applications [8]. Although the biological effects of essential oils or larger groups of phenylpropanoid compounds like flavonoids have been extensively reviewed in previous publications [9,10], a smaller group of volatile phenylpropenes has rarely been discussed to the present date. Hence, this review was focused on the presentation of the latest research regarding the pharmacological effects and potential health benefits of volatile phenylpropenes, revealing the available preclinical and clinical studies, but also mechanistic and toxicity data, where available.

## 2. Literature Search Methodology

This work is a narrative, non-systematic review. Articles were identified through searches in PubMed, Science Direct, Scopus, and Web of Knowledge platforms, covering the interval from 2011 to 2026. The keywords combined the names of the individual phenylpropene compounds with terms describing pharmacological activity. No formal protocol was registered, and no systematic screening or risk-of-bias assessment was performed. Studies were selected to provide representative evidence across the major pharmacological domains, with priority given to recent investigations, isolated compounds, quantitative outcomes, mechanistic studies, and in vivo confirmation where available. All publications included in this review were written in English. Conference abstracts, preprints, and unrelated studies were excluded.

## 3. Chemistry, Biosynthesis, and Natural Sources of Volatile Phenylpropenes

Phenylpropenes, a subclass of phenylpropanoid compounds, are a significant category of plant volatiles, playing a critical role in enhancing the flavor of prominent herbs and spices.

Volatile phenylpropenes, also called alkenyl benzenes, are named after their chemical structure which consists of an aromatic phenyl core and an unsaturated three-carbon propene tail. Potential hydroxy, methoxy, or methylene-dioxy substitutions on the aromatic ring are possible [11]. The most important volatile phenylpropenes are anethole, chavicol, estragole, eugenol, myristicin (methoxy-safrole), and safrole. Cinnamaldehyde, although not an alkenyl benzene in the strict structural sense, is also included in the phenylpropene group as a closely related volatile C6–C3 molecule, with a shared biosynthetic origin. The chemical structures of the most representative volatile phenylpropenes are presented in Figure 1.

Volatile phenylpropenes are biosynthesized via the monolignol pathway from phenylalanine by multiple enzymatic reactions [12]. Initially, phenylalanine is deaminated by phenylalanine ammonia-lyase resulting cinnamic acid, which is further hydroxylated to coniferyl alcohol. Then, an acyltransferase (CFAT) yields coniferyl acetate which is transformed into eugenol by another enzyme, eugenol synthase (EGS), from the short-chain dehydrogenase/reductase (SDR) family. Further, an O-methyltransferase generates methyl-eugenol, but it can also generate para-OH compounds like estragole and anethole from corresponding precursors [12,13]. Cinnamaldehyde is also formed from cinnamic acid by the intervention of 4-coumarate: CoA ligase (4CL) and cinnamoyl-CoA-reductase (CCR). Phenylpropene molecules can be detected as free volatiles or in glycosidic forms [12,13,14].

Volatile phenylpropenes are widely distributed throughout angiosperms, being the major constituents in essential oils from several plant species like basil (*Ocimum basilicum*), clove (*Syzygium aromaticum*), cinnamon (*Cinnamomum* sp.), sassafras (*Sassafras albidum*), nutmeg (*Myristica fragrans*), and fennel (*Foeniculum vulgare*), but can also be found in variable amounts in a range of fresh fruit species including apple, strawberry, and grape [1,5]. Volatile phenylpropenes like eugenol have also been found in essential oils from gymnosperms species like conifers (pine, spruce, larch, and juniper), although rare [15]. The concentration of phenylpropenes in specific plant species may depend on geographical factors, plant varieties, and plant organs (leaves or fruits), but also on the method of extraction [16].

## 4. Pharmacological Effects and Health Applications of Volatile Phenylpropenes

### 4.1. Antimicrobial Effects

In recent decades, the emergence of a significant resistance of pathogenic bacteria and fungi to common antibiotic and antifungal drugs has stimulated the quest for new alternative molecules. In this context, the use of natural compounds like phenylpropene molecules could play an important preventive and therapeutic role [17].

A series of preclinical studies have investigated the antimicrobial effects of phenylpropenes, mostly using in vitro models. Mechanistically, phenylpropenes were found to have several distinct antibacterial and antifungal mechanisms of action, including impairment of the structure of bacterial/fungal membranes, inhibition of bacterial/fungal enzymes, and alteration of bacterial ribosomal protein synthesis [18].

Anethole (trans isomer) was able to inhibit the growth of mupirocin-resistant strains of *Staphylococcus aureus* in an in vitro experiment which used disk diffusion models. The combination of trans-anethole-mupirocin proved to exert significant synergistic effects, improving the efficacy of the antibiotic drug and reducing biofilm formation. The results of the experiment showed that trans-anethole increased the growth inhibition zone from 30.5 to 53.5 mm for bacterial strain *S. aureus* ATCC 29213, susceptible to mupirocin [19]. Moreover, in an ex vivo study which used a human whole-blood model infected with the *Staphylococcus aureus* Newman strain, pretreatment with trans-anethole proved to have significant immunomodulatory effects, upregulating pro-inflammatory cytokines and chemokines like CCL2/MCP-1-CCL4/MIP-1beta (Monocyte Chemoattractant Protein1-Macrophage Inflammatory Protein-1beta), CXCL1 (CXC Motif Chemokine ligand 1), CXCL2 (CXC Motif Chemokine Ligand 2), MMP-8 (Matrix Metaloproteinase-8), and PGLYRP1 (Peptidoglycan Recognition Protein 1) in the plasma, thus improving the immune response of blood cells against the bacteria [20].

The hydroxylated form of chavicol (hydroxychavicol), which is naturally present in high concentrations in the leaves of the *Piper betle,* proved to exert significant antibacterial effects. An in vitro study from 2017 using disk diffusion methods showed that hydroxychavicol had strong antimicrobial effects against *E. coli* and *Salmonella* sp., bacterial species which often cause food-borne diseases with minimal bactericidal concentrations (MBCs) of 20–50 µg/mL [21]. A subsequent study from 2018 confirmed the antibacterial effects of hydroxychavicol against *E. coli* strain K12 BW25113, showing that the tested compound caused damage to bacterial iron sulfur proteins and also to DNA, with bactericidal effects and an MBC value of 500–1000 µg/mL [22].

Cinnamaldehyde, present in the bark and leaves of cinnamon trees, has been proven to exert antibacterial activity against several pathogens like *E. coli*, *Listeria monocytogenes*, *Pseudomonas aeruginosa,* and *Staphylococcus aureus*. Previous studies showed that its’ general mechanism of action is the structural modification of bacterial cell membranes [23], but a more recent study gained additional in-depth data, proving that cinnamaldehyde interfered with glycerophospholipid biosynthesis pathways by targeting phosphatidyl-glycerol and phosphatidyl-ethanolamine, with a subsequent disruption to bacterial cell membrane integrity in the case of *S. aureus* and *E. coli* [24].

Estragole was evaluated in vitro using an ethidium bromide method against two strains of bacteria with efflux pump mechanisms: *Staphylococcus aureus* 1199B and *S. aureus* K2068 with NorA and MepA pumps. The study showed the potential blocking effect of estragole on the bacterial efflux pumps, with augmentation of the antimicrobial efficacy of several associated antibiotics like norfloxacin. The results of the experiment proved that estragole was able to reduce norfloxacin MIC from 322.54 to 256 µg/mL, acting synergically with the antibiotic [25]. Moreover, a recently published study from 2026 showed a significant antimicrobial effect of estragole against bacterial pathogens considered an absolute priority by the World Health Organization (WHO): multi-drug resistant (MDR) and extensively drug resistant (XDR) strains of *E. coli*, *Klebsiella pneumoniae*, *Acinetobacter baumanii,* and *P. aeruginosa*. The results showed that estragole had good antibacterial effects with a minimal inhibitory concentration (MIC) under 256 µg/mL, but it also proved to have synergic effects with meropenem and tobramycin, with a significant reduction in the MIC of up to 16-fold. Thus, the capacity of estragole to become an adjuvant capable of augmenting the efficacy of existing antibiotics against resistant bacteria has been clearly demonstrated [26].

Eugenol has proven to have broad-spectrum antibacterial effects and has been effective against a series of bacterial strains which often cause severe and difficult-to-treat infections. In a study on four different carbapenem-resistant *Klebsiella pneumoniae* isolates, eugenol showed a significant inhibitory effect with a MIC of 0.2 mg/mL. The effect was attributed to the disruption of the bacterial cell membrane and subsequent increase in its permeability, with loss of intracellular matter [27]. Eugenol’s antibacterial mechanism is complex. It alters the integrity of bacterial cell walls, leading to the loss of cytoplasm and intracellular components, but it can also interfere with bacterial protein synthesis. Research has shown that in Gram-negative bacteria, eugenol was able to penetrate the lipopolysaccharide layer and to induce cytotoxic effects in bacterial cells by augmenting the generation of reactive oxygen species [18].

Although volatile phenylpropenes are lipophilic molecules which can easily traverse biological membranes, they could present a degree of chemical instability which could impact the studies aimed at exploring their antimicrobial potential. Therefore, novel formulations using nanoplatforms (nano-emulsions, nanoparticles, or liposomes) could improve their chemical stability and bioavailability. Thus, in a recently published study from 2026, the antibacterial potential of a novel nanoplatform with eugenol combined with limonene was tested both in vitro and in vivo. The in vitro model, which used the disk diffusion assay, exerted significant antibacterial activity with MICs of 8–32 µg/mL, while the in vivo model of pneumonia in rats showed a reduction in bacterial load and bronchiolar irritation [28].

A large research effort was dedicated to the investigation of the ability of phenylpropenes to reduce bacterial resistance to common antibiotics, a phenomenon which poses a significant threat to public health systems. Some phenylpropenes were proven to be capable of inhibiting microbial biofilm formation, with positive implications for the increasingly important fight against nosocomial infections caused by resistant bacteria. Due to their chemical structure, phenylpropene molecules can more easily traverse the biofilm matrix, reaching incorporated bacterial cells that are otherwise protected against regular antibiotic drugs [29]. Also, certain phenylpropenes like eugenol were capable of eliminating the bacterial biofilm present on medical implants, a frequent source of bacterial infections, by reducing the viability of biofilm-forming cells [30].

Specific studies have confirmed that eugenol is able to reduce bacterial virulence and alter biofilm structures. An in vitro study showed that eugenol reduced the production of virulence factors (pyocyanin, violacein, and elastase) and caused a significant inhibition of biofilm production in case of *E. coli* [31]. A newer study from 2019 demonstrated that eugenol attenuated the virulence of *Pseudomonas aeruginosa* by reducing the synthesis of acyl-homoserine lactones (AHLs) as well as pyocyanin and violacein [32]. Moreover, other phenylpropenes like cinnamaldehyde and methyl-eugenol were able to reduce AHLs and AI-2 signaling in *E. coli* and *V. harveyi*, and to inhibit their ability to form biofilm in other clinically important bacteria like *Pseudomonas aeruginosa* and *Proteus mirabilis* [33].

A reduction in quorum sensing (QS) gene expression was another identified mechanism by which phenylpropenes can decrease bacterial virulence and reduce the pathogenicity of bacterial communities [34]. Specifically, eugenol showed an ability to reduce the gene expression of both *las* and *pqs* quorum sensing systems in *E. coli*, but also the expression of *lasI* and *rhIA* QS genes in *Pseudomonas aeruginosa* [31,32]. Also, eugenol downregulated *mecA* and *agrA*, bacterial genes associated with virulence, in the case of methicillin-resistant *S. aureus* isolates from human patients [35]. Recent findings concerning phenylpropenes’ ability to reduce bacterial resistance and virulence are summarized in Figure 2.

The antifungal effect of volatile phenylpropenes has also been investigated. A complex in vitro and in vivo study showed that CIN-102, a mixture containing 86.7% cinnamaldehyde, proved to exert significant antifungal effects against *Madurella mycetomatis*, a fungus causing severe tropical infections. The results of the study showed that CIN-102 presented a MIC_50_ of 128 µg/mL in the in vitro experiment. The tested product also showed promising results in the in vivo experimental model which used infected *Galleria mellonella*, increasing the survival time of the larvae [36]. Another volatile phenylpropene, eugenol, has been intensely researched, with multiple studies showing good efficacy against various *Candida* species like *Candida albicans*, *C. auris*, or *C. gattii*, but also against *Cryptococcus neoformans* [37]. The antifungal effect of eugenol relies on multiple mechanisms. The compound can inhibit ergosterol synthesis in fungal cells, a mechanism resembling antifungal azoles, but it can also alter the functionality of amino acid permease and reduce the virulence factors involved in fungal adhesion to host epithelial cells [38]. Additionally, data obtained from a series of studies showed that eugenol was able to reduce biofilm formation and also hyphal proliferation and filamentation, suggesting a possible role in resistant fungal infections. The effect is particularly valuable given the fact that mature fungal biofilms show reduced ergosterol content, with significant resistance to existing antifungal drugs like azoles [39].

In conclusion, phenylpropenes can significantly inhibit biofilm formation and reduce virulence in both bacteria and fungi. They could become valuable agents capable of mitigating the severity of microbial infections, making them promising adjuvants or drug candidates associated with existing antibiotic or antifungal drugs for improved clinical responses.

### 4.2. Antitumoral Effects

Phenylpropenes are increasingly regarded as potential chemo-preventive agents due to their antitumoral effects, being able to interfere with cell-cycle regulation and cell growth in some solid tumors but also being able to act synergically with existing anticancer drugs [40].

The antitumoral effects of anethole were tested in vitro on gingival cancer cells Ca9–22: the results showed a significant reduction in cell proliferation, with an IC_50_ value of 10 µM. The study proved that anethole modified the expression of 13 genes with roles in the control of the cell cycle, and seven genes which regulate apoptosis [41]. Another study proved that anethole was able to reduce the tumor size in xenograft models of cancer which used the non-small-cell lung cancer (NSCLC) line A549 in BALB nude mice. Treatment with 50 µM pf anethole reduced colony numbers from 200 to less than 100 in the colony formation assay of A549 cells. Anethole acted by a complex mechanism which combined the inhibition of angiogenesis with the modulation of the PI3K/Akt/mTOR pathway and activation of caspase-3, which promotes cancer cell death [42]. Additionally, anethole proved to exert synergic effects with several antitumoral drugs on specific cancer cells. It augmented the effects of doxorubicin on MDA-MB-231 triple-negative breast cancer cells, the effects of lactacystin, a proteasome inhibitor on DU145 prostate cancer cells, and the effects of etoposide, also on MDA-MB-231 cancer cells [43].

The anticancer effect of cinnamaldehyde has also been tested in a variety of experimental models. Haripriya et al. showed, in an in vitro study, a significant inhibitory effect of cinnamaldehyde on HepG2 cells accompanied by the augmentation of caspase-3 activity [44]. Cinnamaldehyde also showed good results against other types of cancer cells. In a study from 2022, cinnamaldehyde induced apoptosis of bladder cancer 5637 cells by the inhibition of ErbB2, HSF1, and LDHA gene expression, acting at concentrations between 0.02 and 0.08 mg/mL [45]. Finally, cinnamaldehyde proved to be effective in more complex experimental models of cancer using in vitro and in vivo techniques. Cinnamaldehyde increased the apoptosis of colon cancer cells HCT116 in a tumor xenograft model in BALB/c nude mice, where a dose of 50 mg/kg produced a significant antitumoral effect obtained by the downregulation of Bcl-2 levels and the upregulation of protein Bax, also acting synergically with oxaliplatin with possible positive implications for cancer chemotherapy [46].

Estragole, a phenylpropene present in large concentrations in essential oil from *Agastache foeniculum*, was also tested for a possible anticancer effect on MCF-7 cancer cells. It showed a dose-dependent cytotoxic effect on cancer cells with IC_50_ values of 74 µg/mL, the activation of caspase-3 being considered the main mechanism of action [47].

Eugenol, another important phenylpropene present in the chemical composition of various essential oils, also proved to have good anticancer effects in vitro. It showed significant antitumoral effects on gingival squamous carcinoma cells (Ca9-22), acting by arresting the cell cycle in the G0/G1 phase by cyclin D1 modulation. The results of the study showed that treatment with eugenol significantly reduced cancer cell proliferation, with an IC_50_ of 200 µM [48]. Additionally, eugenol was tested on triple-negative MDA-MB-231 breast cancer cells and also on SK-BR-3 breast cancer cells, inducing their apoptosis due to the activation of caspases 3 and 9. The results showed that eugenol (4–10 µM) caused a 76.4% inhibition rate in MDA-MB-231 breast cancer cell proliferation and a 68.1% inhibition rate in SK-BR-3 breast cancer cell proliferation [49]. Eugenol was also tested for synergic effects with conventional antitumoral drugs in new formulations with nanoplatforms, characterized by improved stability. In the case of melanoma cells, the association between eugenol and dacarbazine incorporated in liposomes proved to be a significant augmentation to the apoptosis of cancer cells due to the suppression of survivin, an antiapoptotic protein. The inhibition of survivin is particularly important in malignant melanoma cells where it is overexpressed and partially responsible for the resistance of cancer cells against chemotherapy [50].

Myristicin, a major phenylpropanoid from nutmeg (*Myristica fragrans*), demonstrated anticancer effects in vitro against KB cancer cells (oral carcinoma cells), acting by reducing the expression of Bcl-2 genes and subsequently promoting apoptosis [51].

Safrole, a volatile compound present in sassafras, nutmeg, and pepper, was tested for potential anticancer effects on A54 lung cancer cells, showing a pro-apoptotic effect due to the activation of caspase-3 [52]. However, safrole remains a rather controversial molecule due to possible significant adverse reactions like carcinogenicity. According to one study, the presence of safrole in the betel quid prepared from the *Piper betle* vine, consumed in certain parts of Asia, was associated with oral cancers in humans [53].

The diverse antitumoral effects of volatile phenylpropenes and their capacity to act synergically with conventional anticancer drugs highlights their potential to become promising adjuvants or drug candidates for an integrative oncological strategy.

### 4.3. Antioxidant Effects

Oxidative stress can alter important intracellular components like DNA, lipids, or proteins, potentially leading to unwanted cardiovascular events like atherosclerosis which can subsequently cause stroke or myocardial infarction [54]. Other important diseases like diabetes, dyslipidemia, or cancer are also characterized by the involvement of oxidative stress [55]. Reactive oxygen species (ROS), represented by superoxide anions, hydroxyl radicals, and singlet oxygens, are harmful by-products formed during the cell respiratory process and can accumulate during pathological conditions, their removal being a promising therapeutic strategy [56].

Phenylpropene compounds may act as antioxidant agents by a variety of mechanisms. They can reduce ROS formation by inhibiting specific enzymes or chelating metal ions, but they can also scavenge ROS and augment endogenous antioxidant systems. An important antioxidant capability is the ability to chelate Fe^3+^ ions, involved in the generation of hydroxyl radicals [57].

The allyl moiety present in the molecule of eugenol and other phenylpropenes assures their ability to directly scavenge reactive oxygen species like O_2_ by trapping them, while the hydroxyl group is involved in the transfer of electrons or hydrogen atoms which leads to the neutralization of destructive free radicals [58]. Therefore, multiple hydroxyl groups significantly increase antioxidant capacity, critical for avoiding tissue damage produced by reactive oxygen species. Apart from their direct radical-scavenging ability, phenylpropenes can also activate endogenous antioxidant enzymes like superoxide dismutase and glutathione peroxidase, increasing the antioxidant defense of the organism [30,59].

Another antioxidant defense mechanism is represented by the activation of the Nrf2 (nuclear factor erythroid 2-related factor 2) pathway. Nrf2 is a key transcription factor which upregulates important antioxidant proteins at the cellular level with decisive roles in the defense against oxidative stress-induced DNA lesions. Thus, phenylpropenes like cinnamaldehyde and methyl-eugenol could stabilize Nrf2 by inhibiting its degradation mediated by Keap1, allowing its cellular accumulation with potent antioxidant effects [60].

Numerous in vitro assays like ABTS, FRAP, and DPPH, as well as free radical scavenging assays and in vivo models have been used to evaluate the antioxidant potential of phenylpropanoids.

Anethole has strong antioxidant effects due to its propenyl side chain, where the double bond is conjugated with the aromatic ring, enhancing its capacity to delocalize electrons and to be an effective ROS scavenger. In a DPPH assay, essential oil from *Illicium verum* with 92.2% trans-anethole proved to have significant antioxidant effects, with an IC_50_ of 34.6 μg/mL [61].

Cinnamaldehyde was evaluated in vitro as an antioxidant agent in several studies. In liver homogenates originating from Wistar rats, cinnamaldehyde inhibited lipid peroxidation by 11–33% at concentrations ranging from 1 to 3 µM/mL [62]. Another study on human umbilical vein endothelial cells (HUVECs) found that cinnamaldehyde protected the HUVECs against peroxide-induced oxidative stress by the activation of nuclear factor erythroid 2-related factor 2 (Nrf2) [63].

An in vitro study from 2025 tested the antioxidant activity of eugenol, bis-eugenol, and clove oil (with 45–90% eugenol) using DPPH and FRAP techniques. It was found that the compounds (25 μg/mL) neutralized free radicals and reduced ferric ions, with DPPH radical scavenging of around 80% and FRAP values from 200 to 300 μM Fe2+ equivalents [64].

An in vitro study from 2018 showed that methyl-chavicol inhibited DPPH free radicals and reduced lipid peroxidation by 73.08%, with an IC_50_ of 312.50 ± 2.28 μg/mL [65].

Kuang et al. showed that another phenylpropene, methyl-eugenol, was also able to activate Nrf2, protecting the kidneys in an oxidative damage experimental model in mice. The administration of 20 mg/kg methyl-eugenol for 5 days attenuated renal destruction and improved survival rate, reducing mitochondrial lesions in the acute kidney injury induced by a surgical procedure [66].

### 4.4. Anti-Inflammatory Effects

Inflammation is a physiological defense reaction of the body triggered by a mechanical, chemical, or microbial injury. Although it is usually self-limiting, in certain conditions it can become chronic with a significant impact on patients’ quality of life. In typical inflammation, specific immune cells migrate from the blood into the tissues, releasing an array of chemical mediators at the inflammatory site as a result of the activation of key enzymes [8].

Natural products with anti-inflammatory activity, like phenylpropenes, can act directly on the immune cells or inhibit enzymes involved in the development of inflammatory reactions. The capacity of phenylpropenes to mitigate inflammatory processes could offer important therapeutic applications in inflammatory diseases of the digestive tract, kidney injuries, and inflammatory joint diseases, but also in atherosclerosis or metabolic diseases [8,64].

In vivo, anethole—the major constituent present in essential oil from anise and star anise—showed a complex anti-inflammatory effect, characterized by the inhibition of important pro-inflammatory cytokines like tumor necrosis factor-α and interleukin-1 (TNF-α, IL-1), as well as inhibition of the cyclooxygenase (COX) pathway. The oral administration of anethole in Wistar rats, in doses of 125–250 mg/kg, reduced inflammation in two classical experimental models (paw edema and pleurisy induced by carrageenan), and also showed synergistic effects with ibuprofen [67]. Furthermore, in another study, anethole showed a significant anti-inflammatory effect in a model of acetic acid-induced colitis in mice. It reduced the inflammation of the intestinal mucosa, but also the expression of Il-1, TNF-alpha, and TLR4, after being administered for 7 days in doses of 31.25–250 mg/kg [68]. In vitro, the administration of anethole to HIG-82 synovial cells stimulated with H_2_O_2_ reduced the inflammatory reaction, downregulating aquaporin-1 (AQP1) expression and stimulating the protein kinase A (PKA) pathway, inhibiting the enzymes responsible for cartilage degradation [69].

Cinnamaldehyde is another phenylpropene derivative with demonstrated anti-inflammatory effects. It reduced the levels of pro-inflammatory cytokines like TNF-alpha, IL-1, and IL-6 in LPS-stimulated J774A.1 cells, increasing simultaneously the expression of anti-inflammatory molecules like IL-10 [70]. Also, the administration of cinnamaldehyde in doses of 0.45–0.9 mg/kg to endotoxin-induced mice produced a reduction in peripheral levels of IL-1, IL-6, TNF-alpha, and High Mobility Box-1 (HMGB-1) in the treated animals, coupled with weight loss and enlargement of lymphoid organs. Additionally, cinnamaldehyde was able to reduce the activation of NF-kB (Nuclear factor kappa-light-chain-enhancer of activated B cells) and caspase in the spleen and mesenteric lymph nodes of the treated animals, revealing a complex anti-inflammatory mechanism [71].

Eugenol was tested extensively for its anti-inflammatory potential in several in vitro and in vivo experimental models. The administration of eugenol in doses of 5–10 mg/kg in mice prevented the acute inflammatory reaction caused by intratracheal administration of LPS by the inhibition of TNF-alpha and COX-2 expression [72]. In vitro, eugenol was able to reduce the inflammatory reaction in fibroblast-like synoviocytes from patients with rheumatoid arthritis. The data also showed an inhibition of NF-kB and a reduction in the concentration of vascular endothelial growth factor (VEGF), Il-6, and IL-8 [73]. In silico pharmacological studies have confirmed an inhibition of cyclooxygenase-2 and lipoxygenase, with a reduction in synthesis of pro-inflammatory prostaglandins and leukotrienes. Hence, a computational model suggested that eugenol had dual inhibitory activity against COX-2/LOX enzymes and, due to its selectivity for COX-2, that it could be better tolerated by the gastric mucosa compared with conventional NSAIDs; nonetheless, further in vivo studies are needed to confirm the suggested mechanism of action [74].

Estragole was found to exert significant anti-inflammatory effects by inhibiting the NF-kB pathway in an in vitro study using LPS-induced RAW 264.7 cells. More specifically, it inhibited the phosphorylation and degradation of IkBα, preventing the translocation of NF-kB to the nucleus and the subsequent transcription of pro-inflammatory genes. Additionally, estragole was able to inhibit NO synthesis by reducing the expression of inducible nitric oxide synthase (iNOS) [75].

Myristicin has also shown important anti-inflammatory potential in RAW 264.7 macrophages, where it inhibited the synthesis of a variety of pro-inflammatory molecules: IL-6, NO, MIP-1α, and MCP-3 [76]. A molecular docking study also confirmed the capacity of myristicin to non-selectively inhibit COX-2, with subsequent reduction in PGE2 concentration, a prostaglandin responsible for the development of inflammatory reactions and pain [77].

### 4.5. Metabolic Effects

The metabolic effects of natural products have generated large interest due to their potential therapeutic applications, considering the large number of patients with diabetes mellitus and dyslipidemia. The complexity of glucose metabolism offers a variety of potential pharmacological targets for investigational natural antidiabetic molecules. In dyslipidemia, the main task for a potential natural hypolipemic compound would be to reduce the level of total cholesterol and especially LDL, the atherogenic fraction responsible for major vascular events like heart attacks or strokes [78]. A series of molecules of natural origin have been tested for their antidiabetic or hypolipemic potential, including several volatile phenylpropenes [7,9,10].

Phenylpropene compounds could act as antidiabetic agents by multiple mechanisms: inhibition of glucose absorption, augmentation of insulin secretion, or increasing insulin sensitivity. In the digestive tract, enzymes like α-glucosidase and α-amylase degrade complex carbohydrates to smaller and absorbable units like glucose or maltose. The inhibition of the two enzymes could lead to a reduction in absorbed glucose, with potential application in the reduction in postprandial hyperglycemic peaks [78]. In diabetes, increased gluconeogenesis could be responsible for fasting hyperglycemia observed in certain patients. Thus, its inhibition could represent an important mechanism for potential antidiabetic drug candidates [7,11].

Cinnamaldehyde was tested in a complex study for its anti-obesity and antidiabetic effects. It was found that obese mice treated with cinnamaldehyde for five weeks showed improved glucose tolerance, reduced insulin resistance, and increased energy expenditure due to increased expression of insulin receptors. Additionally, a significant reduction in body weight was recorded in the treated mice due to an upregulation of the TRPA1 (transient receptor potential-ankyrin receptor 1) receptors. The study discovered that TRPA1 and ghrelin receptors are co-localized in duodenal neuroendocrine cells and play a role in food intake behavior, thus making them important targets in the development of new drugs for obesity [79]. Additionally, the research of Yang et al. in 2026 suggests that multiple in vitro and in vivo data support the existence of a synergy between cinnamaldehyde and conventional antidiabetic drugs, the complementary mechanisms of action allowing the use of a lower dose of existing drugs and mitigation of their side effects [80].

Eugenol was evaluated in a complex experiment with in vivo and in vitro components regarding its antidiabetic effects. In vitro, eugenol inhibited α-glucosidase, with an IC50 value of 5 mM. Additionally, in the same in vitro experiment, eugenol caused a 58% reduction in advanced glycation end product (AGE) formation, with a IC50 value of 10 mM. In vivo, the administration of 100 mg/kg of eugenol for 2 weeks in STZ-treated mice caused a 38% reduction in blood glucose [81]. In glucose homeostasis, AMPK (adenosine 5′-monophosphate-activated protein kinase) is an enzyme functioning as a central regulatory element in energy metabolism at the cellular level. The activation of AMPK increases insulin sensitivity and causes beneficial effects in type 2 diabetes. Eugenol was proved to activate the AMPK-GLUT4 pathway, increasing insulin sensitivity and reducing insulin resistance in C57BL/6J mice fed with high-fat diet [82]. Another study investigated the effects of eugenol on the lipid profile of rats fed with cholesterol-enriched diet. The compound, administered orally to the rats in doses of 10–100 mg/kg, reduced total cholesterol, LDL, and the atherogenic index by downregulating the hepatic TRPV1 receptor (transient receptor potential vanilloid 1), a novel mechanism for drugs used in dyslipidemia [83].

Another phenylpropene molecule, methyl-chavicol, was able to inhibit pancreatic lipase by 58.12% in vitro, thus possessing the potential to reduce the absorption of dietary fats. A complementary molecular docking technique showed a high binding affinity (−6.1 kcal/mol) for the lipase, confirming the effectiveness of methyl-chavicol [65].

### 4.6. Other Effects

Some representatives from the volatile phenylpropene class proved to exert local anesthetic and analgesic (antinociceptive) effects in specific experimental models. Eugenol was the first phenylpropene derivative with demonstrated local anesthetic and analgesic properties due to its ability to block neuronal voltage-gated sodium channels and to modulate TRPV1 receptors in the rat trigeminal system [84]. Based on these effects and also on its antimicrobial properties, eugenol is widely used in dentistry as zinc oxide–eugenol cement for temporary fillings and root canal sealing [85]. Other phenylpropenes were found to have analgesic and local anesthetic properties in several experimental models. A blocking effect on the neuronal voltage-gated Na channel with local anesthetic-like effects was identified by an in vitro study using anethole [86]. Furthermore, estragole showed promising antinociceptive potential in formalin, hot-plate, and tail-flick tests in rodents due to the modulation of nitric oxide, glutamatergic, and vanilloid pathways [87].

Other studies have investigated the neuroprotective effects of certain phenylpropenes, especially eugenol. An in vitro study focused on the evaluation of the preventive potential of eugenol in Alzheimer’s disease found that the tested compound inhibited cholinesterase but also butyril-cholinesterase, reducing the degradation of acetylcholine, a neurotransmitter deficient in Alzheimer’s. Additionally, eugenol protected neuronal membranes against lipid peroxidation due to its significant antioxidant potential [88]. Moreover, in a model of pilocarpine-induced status epilepticus in mice, eugenol proved to exert neuroprotective effects, reducing the expression of inflammatory cytokines and the formation of the NLRP3 (NOD-LRR-and pyrin domain-containing protein 3) inflammasome in hippocampal neurons [89]. The main aspects of the in vitro and in vivo studies focused on the pharmacological effects of volatile phenylpropenes are presented in Table 1.

## 5. Clinical Studies with Volatile Phenylpropenes

Although a significant amount of promising preclinical data suggest possible therapeutic roles for volatile phenylpropenes, they have been rarely tested in a clinical setting in humans. A randomized placebo-controlled clinical study from 2006, with limited statistical significance, investigated the antidiabetic potential of cloves with a high content in eugenol. According to the study protocol, 36 patients with type 2 DM were divided into four groups and treated orally with different doses of cloves (1–3 g/day, for 30 days), with the results showing a significant decrease in blood glucose but also in triglycerides and LDL at the end of treatment [90].

The antiplaque and antigingivitic effects of a mouth rinse containing essential oil from cloves (rich in eugenol), tea tree oil, and basil was tested in a small, randomized, double-blind clinical trial that enrolled 50 patients. The mouth rinse was applied locally in the oral cavity twice a day (10 mL) for 21 days. At the end of the trial, the treated patients (n = 25) presented a significant reduction in gingivitis, with a decrease in microbial colony-forming units (CFUs) [91].

Another volatile phenyl propene of clinical interest is cinnamaldehyde. Although the antidiabetic potential of cinnamon has been clinically tested in multiple clinical trials, it has not been possible to relate the clinical effects to cinnamaldehyde only due to the complex chemical composition of the spice, which includes other molecules with antidiabetic effects.

Nevertheless, isolated cinnamaldehyde was clinically tested for use on oral mucosa in the treatment of oral fungal infections with *Candida* sp. A phase I clinical trial from 2022 enrolled 35 patients treated with an ointment containing cinnamaldehyde (200, 300, and 400 mcg/mL), applied daily for 15 days, in the oral cavity. At the end of the trial, treatment with the cinnamaldehyde ointment caused a significant reduction in *Candida* sp. CFUs, the cytology analysis showing no cell modifications of the oral mucosa [92].

Cinnamaldehyde was also tested in 2025 by Molania et al. in aphthous stomatitis in a small, randomized, double-blind clinical trial which enrolled 44 patients. Cinnamaldehyde was administered locally, three times a day, as mucosal adhesive patches. After 3, 5, and 7 days of treatment, it was found that ulceration size and mean pain intensity were significantly reduced in the cinnamaldehyde group compared to the placebo group [93].

The major limitation of the clinical studies evaluating the potential beneficial effects of phenylpropenes was low statistical significance due to the limited number of patients enrolled in the trials. Additionally, in some clinical studies, essential oils rich in phenylpropenes were used. Therefore, the results of these studies should be interpreted with caution, considering the complex chemical composition of the essential oils with other active substances which could contribute to the overall effect. Additional clinical trials with isolated volatile phenylpropenes, with a superior statistical significance and better design, are warranted to confirm their possible role in human therapy.

## 6. Structure-Activity Relationships (SARs)

A limited number of studies have explored the structure-activity relationships (SARs) of volatile phenylpropenes, investigating primarily antimicrobial and antioxidant effects.

The benzene ring coupled with an alkenyl side chain is the backbone structure present in the molecules of volatile phenylpropenes. This core structure is responsible for their lipophilic nature, being capable of infiltrating into microbial lipid bilayers, disrupting membrane integrity, and facilitating leakage of intracellular components, with a subsequent loss in microbial cell viability [94].

Other functional groups present in the molecules of volatile phenylpropenes may also contribute to their overall antimicrobial effect. In the case of cinnamaldehyde, the unsaturated aldehyde group attached to the benzene ring can react with electrophilic structures such as microbial enzymes, leading to microbial inhibition [95].

Eugenol presents a hydroxyl and a methoxy group attached to the benzene ring. According to one study, the hydroxyl moiety may facilitate hydrogen bonding with bacterial proteins present in the cell wall, increasing membrane permeability and loss of intracellular components, while the methoxy group is involved in the augmentation of eugenol’s lipophilicity [30,94,96]. In the case of estragole (methyl-chavicol), the methoxy group in the para position on the benzene ring acts by increasing lipophilicity, facilitating a better integration of the compound in microbial lipid membranes [94].

For myristicin and safrole, the presence of a methylenedioxy group augments the ability of the compound to interact with nucleophilic sites of microbial enzymes through the alteration of microbial protein synthesis [94,97].

The antioxidant effect of volatile phenylpropenes directly depends on the hydroxyl groups, which are able to transfer electrons in order to neutralize free radicals and to reduce destructive oxidative processes. Thus, the presence of multiple hydroxyl groups on the benzene ring increases its ability to donate hydrogen and the magnitude of any antioxidant effect [98].

The position of the double bond in the alkenyl side chain could also play a role in the antioxidant effect of phenylpropenes. According to one study, anethole proved to exert a superior antioxidant effect, when compared to estragole, due to the conjugation of its alkenyl double bond with the aromatic ring [99].

The versatile chemical structure of volatile phenylpropenes could serve as a backbone for the synthesis of new derivatives with improved physicochemical properties, but further research is needed to better understand their potential role in therapy.

## 7. Safety Profile of Volatile Phenylpropenes

Aromatic herbs with a high content of volatile phenylpropenes have been widely used by humans for culinary purposes. Moreover, individual phenylpropenes play an important role in the cosmetic industry and are currently used as dental or oral products. In cases of specific phenylpropenes or essential oils rich in phenylpropenes, health regulatory authorities have approved their human use. Thus, eugenol has gained the status of a safe food additive, being generally recognized as a safe substance (GRAS) according to FDA documents. Also, the FDA has approved the use of clove oil as a flavor substance both in the food and cosmetic industries [100].

However, the designation of specific phenylpropenes as safe substances is valid only for the use of small amounts. When used in large doses, or in patients with allergic history, occasional reports of significant adverse effects of phenylpropenes have been published. A significant overdose of eugenol-rich clove oil resulted in severe hepatotoxicity in children, with signs of intoxication which resembled paracetamol hepatic toxicity [101]. Also, immunologic adverse effects of eugenol were also documented in the case of eugenol-based dental products which caused skin irritation, allergic dermatitis, or skin ulcers [102].

In case of other phenylpropenes like estragole, safrole, myristicin, and methyl-eugenol, significant safety concerns have been raised by several studies. Metabolic particularities with potential harmful consequences have been identified for estragole. In phase 2 metabolic reactions, estragole can suffer sulfoconjugation reactions catalyzed by sulfotransferase (SULT), leading to the formation of a toxic metabolite, 1′-sulfoxyestragole, capable of forming DNA adducts and causing genotoxic and carcinogenic effects in rodents [103]. However, the administration of estragole in doses less than 50 mg/kg and its association with SULT inhibitors can avoid possible adverse reactions [103].

Safrole is another phenylpropene with reports of toxic effects. The administration of a diet with a 0.1–0.5% content of safrole to rats for 13 weeks caused hepatocellular carcinomas [104]. In humans, the frequent chewing of betel quid in SE Asia caused an increase in the incidence of oral squamous cell carcinoma, due to its high safrole content [105].

Myristicin is present in high concentrations in nutmeg, also reported in several toxicological studies. The administration of high doses of nutmeg (1–4 g/kg) to mice for 7–14 days caused significant hepatotoxic effects. In humans, 10–15 g of nutmeg, equivalent to 400 mg myristicin, causes neurotoxic effects, leading to anxiety and mental confusion due to a possible GABA receptor antagonism [97]. Furthermore, it has been reported that myristicin can be converted in humans by CYP1A1 into an amphetamine-like metabolite, 3-methoxy-4,5-methylenedioxy-amphetamine (MMDA), with psychoactive effects [97].

Methyleugenol has also generated toxicological reports due to its extensive use in agricultural products and insecticides. A 28-day study in rats where methyleugenol was administered by inhalation proved to alter blood erythrocytes but also to increase hepatic aminotransferases, suggesting a possible hepatotoxic effect [106].

In conclusion, while the majority of phenylpropenes are regarded as safe compounds, for estragole, safrole, myristicin, and methyl-eugenol, long-term ingestion and the use of high doses could cause significant adverse effects. Therefore, the EU Scientific Committee for Food (EU-SCF) has specified a maximum level for safrole of 1 mg/kg in foods and beverages, which is also considered a class 2B carcinogen by the International Agency for Research on Cancer (IARC). For the other compounds, no exposure limits have been established [107].

## 8. Pharmacokinetic and Translational Limitations

Volatile phenylpropenes are lipophilic compounds that are rapidly absorbed but extensively metabolized, particularly by glucuronidation and sulfation, which limits their systemic bioavailability both in humans and laboratory animals [1,7]. Moreover, the inherent volatility of phenylpropenes and their low water-solubility and potential chemical instability may complicate the production of adequate pharmaceutical formulations [28,108]. Hence, the use of nano-based strategies could improve both the chemical stability and pharmacokinetic profile of volatile phenylpropenes. Various nanoplatforms have been tested, including liposomes, nano-emulsions, dendrimers, and nanoparticles of biodegradable polymers, showing an increased bioavailability and a more efficient delivery of phenylpropene molecules. However, the evidence from these studies remains limited and mainly preclinical. Further investigations should compare the concentrations of active compounds in specific tissues and assess additional pharmacokinetic parameters of novel nano-formulations [28,50,108].

Another limitation of the studies investigating the pharmacological effects of volatile phenylpropenes is the lack of in-depth research on dose–response relations. In particular preclinical studies [25,80], the required phenylpropene concentration is unlikely to be reached systemically in humans after oral administration. Consequently, the effects observed at high concentrations in cell-based assays should be regarded mainly as evidence of biological plausibility, with additional studies being needed to clarify their therapeutic potential.

The smallest translational gap is expected for applications in which volatile phenylpropenes could act locally, including mucosal and other topical formulations, dental products, antimicrobial coatings, and anti-biofilm applications on medical devices. Dental materials such as zinc oxide–eugenol cements for temporary fillings are already available on the market [85]. New modern formulations like dental gels or biodegradable dental films with eugenol and other volatile phenylpropenes are very promising, providing superior physicochemical characteristics and a controlled release profile of the active substance [109].

## 9. Conclusions

Volatile phenylpropenes are a promising group of natural molecules with various applications in agricultural, cosmetic, and biomaterials industries. Their antimicrobial, antitumoral, antioxidant, anti-inflammatory, analgesic, and metabolic effects have been investigated in a multitude of preclinical in vitro and in vivo studies. The research has generated promising results, which may suggest possible roles as adjuvants or drug candidates in the treatment of infectious, inflammatory, cardiovascular, or metabolic diseases. However, the molecular mechanisms of action and the safety profiles have not been fully elucidated for all the molecules belonging to volatile phenylpropenes. Further pharmacokinetic, toxicological, and especially clinical studies are needed to better understand their possible role in therapy.

## Figures and Tables

**Figure 1:**
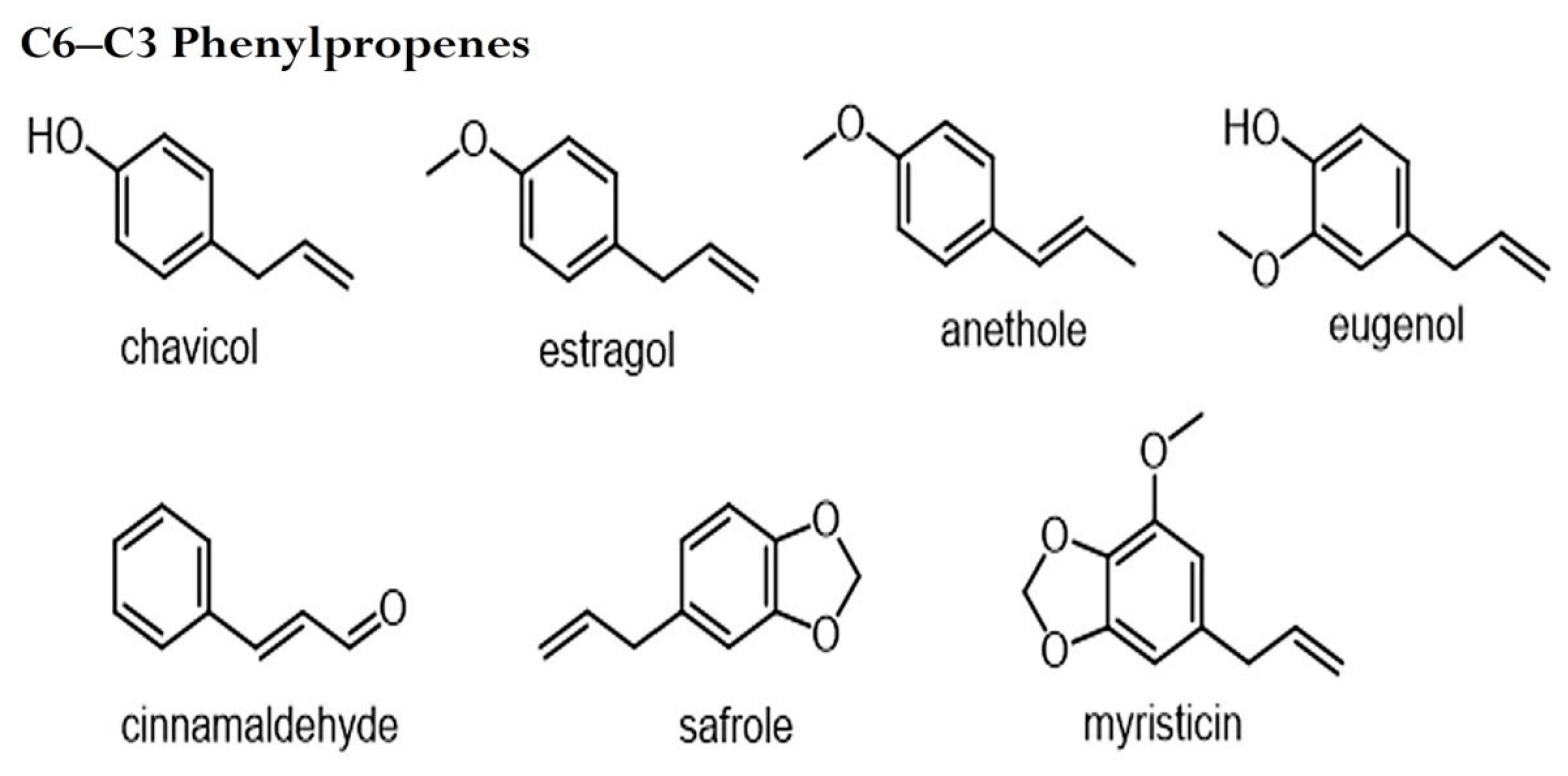
Main naturally occurring volatile phenylpropenes.

**Figure 2:**
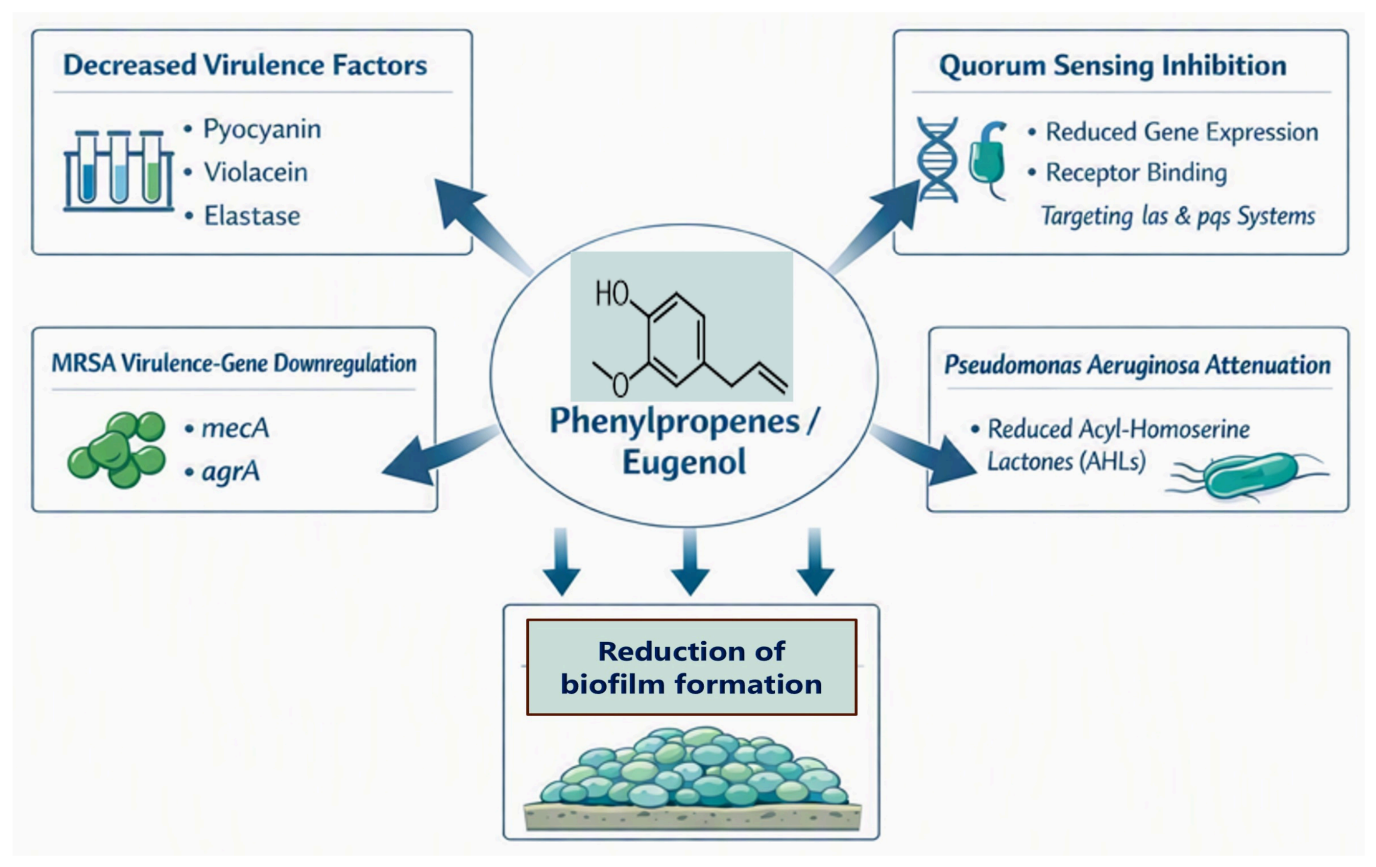
Schematic representation of phenylpropene-mediated inhibition of bacterial biofilm formation and virulence factors.

**Table 1:** Preclinical studies (in vitro and in vivo) investigating the potential pharmacological effects of volatile phenylpropenes.

No.	Substances/Experimental Model	Main Findings	Reference
	**Antimicrobial effects**		
1.	Anethole (trans isomer)/In vitro disk diffusion method	Antibacterial effect against *S. aureus*/synergistic effect with mupirocin	Kwiatowski et al., 2019 [19]
2.	Anethole (trans isomer)/Ex vivo human whole-blood model	Improved immune response of human blood cells in *S. aureus* infection	Kwiatowski et al., 2023 [20]
3.	Hydroxychavicol/In vitro disk diffusion method	Antibacterial effects against *E. coli* and *Salmonella* sp. with an MBC of 20–50 µg/mL	Thamaraikani et al., 2017 [21]
4.	Hydroxychavicol/In vitro disk diffusion method	Antibacterial effect against *E. coli* strain K12 BW25113 with an MBC of 500–1000 µg/mL	Singh et al., 2018 [22]
5.	Cinnamaldehyde/In vitro bacterial cultures/PCR	*S. aureus* and *E. coli* inhibition/disruption of bacterial glycerophospholipid biosynthesis	Pang et al., 2021 [24]
6.	Estragole/In vitro disk diffusion method/ethidium bromide method	Antibacterial effect against *S. aureus*/inhibition of bacterial NorA and MepA efflux pumps	Da Costa et al., 2021 [25]
7.	Estragole/In vitro microdilution method	Inhibition of multiple MDR/XDR bacteria with MICs under 256 µg/mL/synergistic effect with meropenem	Araque et al., 2026 [26]
8.	Eugenol/In vitro agar dilution method/TEM method	Antibacterial effect against four strains of carbapenem-resistant *Klebsiella pneumoniae*/biofilm inhibition	Qian et al., 2020 [27]
9.	Eugenol/limonene nano-emulsion/In vitro disk diffusion method/In vivo pneumonia model in rats	Antibacterial effect against *Pseudomonas aeruginosa* with an MIC of 8–32 µg/mL/Inhibition of bronchial irritation and alveolar remodeling in infected rats	Elwakil et al., 2026 [28]
10.	Eugenol/In vitro disk diffusion method/PCR	Inhibition of biofilm formation for *Pseudomonas aeruginosa*/reduction in expression of QS genes (*las*, *pqs*)	Lou et al., 2019 [32]
11.	Eugenol/In vitro agar well diffusion method/PCR	Inhibition of MRSA strains collected from human patients/reduction in expression of QS genes (*mecA*, *agrA*)	Vinodhini et al., 2025 [35]
12.	CIN-102 (86.7% cinnamaldehyde)/In vitro agar well diffusion method/In vivo infected Galleria mellonella larvae	Significant antifungal effect against *Madurella mycetomatis* with an MIC50 of 128 µg/mL/increase in survival time of infected larvae	Konings et al., 2021 [36]
13.	Eugenol/In vitro agar plate test/crystal violet-based method	Fungicidal activity against *Candida albicans*/reduction in virulence factors responsible for fungal adhesion	Pootong et al., 2022 [38]
	**Antitumoral effects**		
14.	Anethole/In vitro Ca9–22 gingival cancer cells/MTT assay/Pi/Annexin V assay/PCR	Reduction in proliferation of Ca9-22 cells with an IC50 of 10 µM/downregulation of multiple genes involved in the control of the cell cycle	Hammache et al., 2024 [41]
15.	Anethole/In vivo NCSLC xenograft in BALB nude mice	Reduction in tumor size in mice/modulation of the PI3K/Akt/mTOR pathway/activation of caspase-3	Kumar et al., 2023 [42]
16.	Cinnamaldehyde/In vitro HepG2 cells/MTT assay	Reduction in proliferation of HepG2 cells/activation of caspase-3	Hariprya et al., 2022 [44]
17.	Cinnamaldehyde/In vitro bladder cancer 5637 cell line/MTT assay	Increased apoptosis rate of 5637 cells/reduction in gene expression for ErB2, HSF1, and LDHA	Aminzadeh et al., 2022 [45]
18.	Cinnamaldehyde/In vivo HCT116 cancer cell xenograft in BALBc nude mice	Reduction in tumor size in mice/downregulation of Bcl-2/upregulation of Bax	Wu et al., 2019 [46]
19.	Estragole/In vitro MCF-7 cancer cells/MTT assay	Reduction in proliferation of MCF-7 cells/activation of caspase-3	Lashkari et al., 2020 [47]
20.	Eugenol/In vitro Ca9-22 squamous cell carcinoma/MTT assay	Reduction in proliferation of Ca9–22 cells/cell-cycle arrest at G0/G1 phase	Issa et al., 2024 [48]
21.	Eugenol/In vitro triple-negative MDA-MB-231 and SK-BR-3 breast cancer cells/MTT assay	Reduction in proliferation of MDA-MB-231 cells/activation of caspases 3 and 9	Abdullah et al., 2018 [49]
22.	Myristicin/In vitro KB cancer cells/MTT assay	Reduction in proliferation of KB cells/reduction in gene expression for Bcl-2	Rengasamy et al., 2018 [51]
	**Antioxidant effects**		
23.	Anethole/In vitro DPPH assay	Significant antioxidant effect with an IC50 of 34.6 μg/mL	Luis et al., 2019 [61]
24.	Cinnamaldehyde/In vitro H_2_O_2_-treated HUVECs	Protection of HUVECs by translocation of Nrf2 to the nucleus	Kim et al., 2020 [63]
25.	Eugenol/In vitro DPPH and FRAP	Significant antioxidant effect with FRAP values from 200 to 300 μM Fe2+ equivalents	Pires Costa et al., 2025 [64]
26.	Methyl-chavicol/In vitro DPPH assay	Antioxidant effect with an IC_50_ of 312.50 ± 2.28 μg/mL	Santos et al., 2018 [65]
27.	Methyleugenol/In vivo oxidative kidney damage in mice	Kidney protection/Nrf2 activation	Kuang et al., 2023 [66]
	**Anti-inflammatory effects**		
28.	Anethole/In vivo paw edema and pleurisy induced by carrageenan in rats	Reduction in inflammatory edema/TNFα inhibition/synergistic effect with ibuprofen	Wiśniewski-Rebecca et al., 2015 [67]
29.	Anethole/In vivo acetic acid-induced colitis in mice	Reduction in intestinal mucosa inflammation/decrease in Il-1 and TNFα	Ghasemi-Dehnoo et al. 2022 [68]
30.	Anethole/In vitro H_2_O_2_-stimulated HIG-82 synovial cells	Reduction in inflammatory reaction/downregulation of AQP1, stimulation of the PKA pathway	Huang et al., 2024 [69]
31.	Cinnamaldehyde/In vitro LPS-stimulated J774A.1 cells	Reduction in inflammatory markers (TNFα, IL-1, IL-6)	Pannee et al., 2014 [70]
32.	Cinnamaldehyde/In vivo endotoxin-induced mice	Reduction in peripheral level of TNFα, IL-1, IL-6/inhibition of NF-kB in lymph nodes	Lee et al., 2018 [71]
33.	Eugenol/In vivo intratracheal LPS administration in mice	Reduction in airway inflammation in eugenol-treated animals/inhibition of TNFα and COX	Huang et al., 2015 [72]
34.	Eugenol/In vitro fibroblast-like synoviocytes (FLSs) from humans with rheumatic arthritis	Reduction in inflammatory response/decrease in VEGF, IL-8, IL-6/inhibition of NF-kB	Wang et al., 2022 [73]
35.	Estragole/In vitro LPS-induced RAW 264.7 cells	Reduction in inflammatory response/NF-kB and iNOS inhibition	Roy et al., 2018 [75]
36.	Myristicin/In vitro LPS-induced RAW 264.7 cells	Reduction in inflammatory response/Inhibition of IL-6, NO, MIP-1α	Lee and Park, 2011 [76]
	**Metabolic effects**		
37.	Cinnamaldehyde/In vivo ob/ob mice	Improved glucose tolerance, reduction in body weight/increased expression of TRPA1 receptors in digestive neuroendocrine system	Camacho et al., 2015 [79]
38.	Eugenol/In vitro BSA-AGE method/In vivo STZ-induced diabetes in mice	Reduction in AGE formation with an IC_50_ of 10 mM/significant hypoglycemic effect in diabetic mice	Singh et al., 2016 [81]
39.	Eugenol/In vivo C57BL/6J mice fed a high-fat diet	Hypoglycemic effect, reduction in gluconeogenesis in the liver/modulation of AMPK in rat hepatocytes	Jeong et al., 2014 [82]
40.	Eugenol/In vivo rats fed a cholesterol-rich diet	Reduction in total cholesterol, LDL, and the atherogenic index/downregulation of TRPV1 receptor	Harb et al., 2019 [83]
41.	Methylchavicol/In vitro pancreatic lipase	Inhibition of pancreatic lipase by 58.12%/inhibition of lipid peroxidation	Santos et al., 2018 [65]
	**Other effects**		
42.	Anethole/In vitro dorsal root ganglion neurons	Local anesthetic activity/blockage of voltage-gated sodium channels	Moreira Junior et al., 2024 [86]
43.	Eugenol/In vivo nociception models in rats	Antinociceptive effect/inhibition of voltage-gated sodium channels/modulation of TRPV1 receptors	Park et al., 2009 [84]
44.	Estragole/In vivo nociception models in rodents	Antinociceptive effect/modulation of nitric oxide, glutamatergic, and vanilloid pathways	Sousa da Costa et al., 2022 [87]
45.	Eugenol/In vitro AchE assay	Inhibition of AchE and BchE/antioxidant effects	Adefegha et al., 2021 [88]
46.	Eugenol/In vivo pilocarpine-induced epilepsy	Neuroprotective effects/inhibition of NLRP3 inflammasome in hippocampal neurons	Zhu et al., 2023 [89]

Abbreviations: MBC—minimal bactericidal concentration; MDR/XDR—multi-drug resistant/extensively drug resistant; MIC—minimal inhibitory concentration; QS—quorum sensing; Nrf2—nuclear factor erythroid 2-related factor 2; TNFα—tumor necrosis factor-α; AQP1—aquaporin-1; NF-kB—nuclear factor kappa-light-chain-enhancer of activated B cells; VEGF—vascular endothelial growth factor; TRPA1—transient receptor potential-ankyrin receptor 1; AchE—acetylcholinesterase; BchE—butirylcholinesterase; NLRP3—NOD-LRR-and pyrin domain-containing protein 3.

## Data Availability

No new data were created or analyzed in this study. Data sharing is not applicable to this article.
